# New Data on Vaccine Antigen Deficient *Bordetella pertussis* Isolates

**DOI:** 10.3390/vaccines3030751

**Published:** 2015-09-14

**Authors:** Valérie Bouchez, Nicolas Hegerle, Francesco Strati, Elisabeth Njamkepo, Nicole Guiso

**Affiliations:** 1 Molecular Prevention and Therapy of Human Diseases, Institut Pasteur, 25 rue du Dr Roux, Paris 75015, France; E-Mails: nhegerle@gmail.com (N.H.); francesco.strati@fmach.it (F.S.); elisabeth.njamkepo-nguemkam@pasteur.fr (E.N.); nicole.guiso@pasteur.fr (N.G.); 2 URAS-CNRS 3012, Paris 75015, France

**Keywords:** *Bordetella pertussis*, pertactin, filamentous hemagglutinin, vaccine antigen production deficience

## Abstract

Evolution of *Bordetella pertussis* is driven by natural and vaccine pressures. Isolates circulating in regions with high vaccination coverage present multiple allelic and antigenic variations as compared to isolates collected before introduction of vaccination. Furthermore, during the last epidemics reported in regions using pertussis acellular vaccines, isolates deficient for vaccine antigens, such as pertactin (PRN), were reported to reach high proportions of circulating isolates. More sporadic filamentous hemagglutinin (FHA) or pertussis toxin (PT) deficient isolates were also collected. The whole genome of some recent French isolates, deficient or non-deficient in vaccine antigens, were analyzed. Transcription profiles of the expression of the main virulence factors were also compared. The invasive phenotype in an *in vitro* human tracheal epithelial (HTE) cell model of infection was evaluated. Our genomic analysis focused on SNPs related to virulence genes known to be more likely to present allelic polymorphism. Transcriptomic data indicated that isolates circulating since the introduction of pertussis vaccines present lower transcription levels of the main virulence genes than the isolates of the pre-vaccine era. Furthermore, isolates not producing FHA present significantly higher expression levels of the entire set of genes tested. Finally, we observed that recent isolates are more invasive in HTE cells when compared to the reference strain, but no multiplication occurs within cells.

## 1. Introduction

*Bordetella pertussis* is the etiologic agent of the highly contagious human respiratory disease whooping cough, or Pertussis. Widespread introduction of vaccination of young children in the 1950s with whole-cell pertussis (wP) vaccines dramatically reduced morbidity and mortality of the disease in children. However, three decades after introduction of intensive vaccination, a change of transmission of the disease was observed; the child-to-child transmission pattern being replaced by an adult/adolescent-to-newborn transmission. This change, due to waning natural or vaccine-induced immunity, called for the implementation of booster vaccination. The high reactogenicity of wP vaccines prevented their use for that purpose. The need for less reactogenic vaccines for use in infants and booster vaccinations conducted to the development of acellular pertussis (aP) vaccines, containing one to five purified and detoxified *B. pertussis* virulence antigens. Several trials demonstrated these aP vaccines to be efficacious and less reactogenic than wP vaccines, allowing their introduction for booster immunization of adolescents and adults [1]. Although pertussis vaccines dramatically reduced the mortality and morbidity of whooping cough in newborns, epidemics still occur in highly-vaccinated areas. Those present a cyclic pattern and huge epidemics occurred in 2012 and 2013 in aP-vaccinated areas [2]. Mathematical modeling based on recent data found evidence of a lower efficacy, as well as shorter duration of protection, of aP vaccines as compared to wP vaccines ([3,4]). Other hypotheses for the higher magnitude of the last cycle include increased awareness of the disease, introduction of more sensitive but maybe less specific biological diagnosis, differences between aP and wP induced immune responses, but also recent evolution of *B. pertussis* species due to immunological pressure ([1,2])*.*

According to Parkhill *et al.* [5] and Diavatopoulos *et al.* [6], *B. pertussis* evolved from *B. bronchiseptica* to become the specialized restricted human pathogen observed today. The introduction of vaccination applied a new selective pressure to the circulating *B. pertussis* populations that, first, had to evolve in response to wP vaccines world-wide, and now to aP vaccines in high income countries only. Changes in *B. pertussis* circulating isolates were already described under wP vaccine pressure using different typing techniques and whole genome sequencing. Allelic variations were observed in genetic regions of antigenic relevance such as in *prn* (17 alleles), encoding the adhesin pertactin (PRN), *ptxP* (20 alleles), the promoter of the pertussis toxin operon encoding pertussis toxin (PT), *ptxA* (11 alleles), encoding the subunit 1 (S1) of PT, *fim2* (two alleles) and *fim3* (six alleles) encoding the fimbrial proteins Fim2 and Fim3 respectively ([1,6,7,8,9]). However, these changes did not impact the effectiveness of efficacious wP vaccines [1]. Isolates collected during the pre-vaccine era were mainly harboring *prn1*, *ptxA2*, *ptxP1* alleles but since the introduction of wP vaccination, isolates harboring *prn2*, *ptxA1*, and *ptxP3* alleles are predominantly circulating. Only two alleles have been described so far for *fhaB* encoding filamentous hemagglutinin (FHA) [10], a major adhesin involved in colonization. Finally, no polymorphism has been detected in the C-terminal RTX region of *cyaA*, encoding adenylate cyclase-hemolysin (AC-Hly) [11], a major toxin for *B. pertussis*’ pathogenicity. Aside from the major virulence factors described above, whole genome sequencing identified an accumulation of SNPs in *B. pertussis* across the different vaccination periods and some of them were associated with *ptxP3* isolates that emerged as a response to wP vaccines ([12,13,14,15]).

While the most observable genetic evolutions in wP vaccine era were allelic and antigenic variations, the introduction of aP vaccines led to the increased circulation of clinical isolates not producing PRN, a phenotype observed in different countries immunizing with aP vaccines [16]. We previously showed that the lack of PRN did not impact the virulence of *B. pertussis* in the murine model of respiratory infection or in humans by comparing clinical symptoms in infants less than six months of age ([8,17,18]). The lack of this adhesin does not seem to impair the transmission of *B. pertussis* or the colonization of its human host as these isolates are increasingly collected in the human population. However, we reported that these isolates present a better fitness in an aP vaccinated background [19] and this observation was also recently suggested in the United States [20]. Despite the obvious loss of PRN by circulating *B. pertussis* isolates, we were also recently able to show that isolates collected in 2012–2013 present specific cytotoxic phenotypes in presence of polyclonal antibodies [19], suggesting that there is more to *B. pertussis*’ evolution under aP vaccine pressure than what catches the eye. Moreover, few isolates not producing FHA were also reported in France [8] and have been collected under different vaccine eras.

It, thus, seems important to further analyze such vaccine antigen deficient isolates as well as isolates producing all the vaccine antigens currently circulating. For these reasons, we selected recent French clinical isolates producing or not PRN and/or FHA that were previously used in different models [19], to further determine their genomic characteristics, analyze their virulence factor gene transcription profile, and finally evaluate their invasive phenotype in a human tracheal epithelial (HTE) cellular model of infection.

## 2. Materials and Methods

### 2.1. Bordetella pertussis Isolates and Growth Conditions

We chose the Tohama strain as reference, two isolates producing all virulence-associated proteins, two PRN non-producing isolates (PRN−) but harboring a different *ptxP* allele, and two isolates non-producing for either FHA (FHA−) or FHA and PRN (FHA−/PRN−) also harboring a different *ptxP* allele (Table 1). All isolates were grown at 37 °C for 72 h on Bordet-Gengou Agar (BGA) supplemented with 15% defibrinated sheep blood and subcultured in the same medium for 24 h.

For transcriptomic experiments, bacteria were grown at 37 °C in Synthetic Stainer Scholte (SS) medium [21] starting from an OD_650_ of 0.2 and reaching OD_650_ of 1 corresponding to mid-log growth phase representative of expression levels observed during the whole exponential growth phase [22]. Such growth conditions allow the analysis of samples in a systematic, reproducible and standardized manner.

**Table 1:** Characteristics of isolates.

Name	Year of Collection	*ptxP*	*ptxA*	*prn*	*fim2*; *fim3*	Sero typing	Western Blot	Reference
Tohama	1950	1	2	1	2-1; 3-1	2	+	[8]
CIP1672	1954	1	1	1	2-1; 3-1	2	FHA−	[8]
FR4624	2009	3	1	2	2-1; 3-2	3	PRN−/FHA−	[8]
FR4684	2010	1	2	1	2-1; 3-1	3	PRN−	[19]
FR4929	2011	21	1	2	2-1; 3-2	3	+	[19]
FR5133	2012	3	1	2	2-1; 3-2	3	+	[19]
FR5187	2012	3	1	2	2-1; 3-1	3	PRN−	[19]

### 2.2. DNA Preparation

For whole genome sequencing, genomic DNA was prepared using the Genomic-tip 500/G anion-exchange columns (Qiagen, Hilden, Germany), according to the manufacturer’s recommendations.

### 2.3. Whole Genome Sequencing (WGS)/Single Nucleotide Polymorphism (SNP) Analysis

Data have been deposited in the European Read Archive: Study Accession Number PRJEB9559. WGS was performed by the Genomic Platform of the Pasteur Institute. Illumina library preparation and sequencing followed standard protocols developed by the supplier (NEBNext Ultra DNA Library Prep Kit for Illumina, New England BioLabs, Evry, France). Briefly, genomic DNA was sheared by sonication, and sheared fragments were end-repaired and phosphorylated. Blunt-end fragments were A-tailed, and sequencing adapters were ligated to the fragments. Inserts were sized using Agencourt AMPure XP Beads (±500 bp; Beckman Coulter Genomics, Brea, CA, USA) and enriched using 10 cycles of PCR before library quantification and validation. Hybridization of the library to the flow cell and bridge amplification was performed to generate clusters, and paired-end reads of 100 cycles were collected on a HiSeq 2000 instrument (Illumina). After sequencing was complete, image analysis, base calling, and error estimation were performed using Illumina Analysis Pipeline version 1.7. Raw sequence files were filtered using Fquality tool (a read-quality filtering software developed by N. Joly from Biology IT Center, Institute Pasteur, France [23]). Mapping *vs.* the reference genome (*B. pertussis* Tohama genome NC_002929) and SNPs detection was done using GALAXY platform (CLC Assembly Cell v4.2 (CLC Bio, Aarhus, Denmark); snpEff (http://snpeff.sourceforge.net/SnpEff.html#citing). SNPs were concatenated using SynTView (http://genopole.pasteur.fr/SynTView/), alignment was done using multalin (http://multalin.toulouse.inra.fr/multalin/) and the phylogenetic tree was constructed using Quicktree (http://mobyle.pasteur.fr/cgi-bin/portal.py?#forms::quicktree). 

### 2.4. RNA Preparation

RNA was extracted from two independent biological replicate for each isolate. Bacteria were centrifuged after adding 19% absolute ethanol and 1% phenol at 8000 rpm, 10 min, 4 °C and cell pellets stored at −80 °C until RNA extraction. Total RNA was extracted with Trizol^®^ method (adapted from [24]). Briefly, cell pellet was re-suspended and lysed in 1 ml of Trizol^®^ (Invitrogen, Carlsbad, CA, USA). The mixture was centrifuged at 13,000 rpm for 15 min at 4 °C and the upper phase containing the RNA was then recovered. The RNA sample was further purified by chloroform/isoamyl alcohol extraction, ethanol precipitation and later submitted to a DNase treatment (Ambion, Carlsbad, CA, USA). RNA integrity and quality was checked on 1% agarose gel TAE 1X and quantified with a NanoDrop^®^ spectrophotometer.

### 2.5. Transcriptomic Analyses of Targeted Virulence Factors

Amplification of targeted genes (*ptxA*, *fhaB*, *prn* and *cyaA*) and reference gene (*recA*) were performed in triplicate for each biological replicates using the LightCycler^®^ RNA Amplification Kit SYBR Green I and the LightCycler^®^ 480 II instrument (Roche Diagnostics GmbH, Mannheim, Germany), with a one-step RT-PCR cycle and amplicon detection based on SYBR Green I fluorescence. Primers were designed using LC Probe design software and are listed in Table 2. Standard curves were made using concentration ranges from 0.05 to 500 ng of RNA and amplification specificity of each targeted gene was checked by melting curve analysis. Efficiency and reliability of PCR amplifications were calculated using the LightCycler^®^ 480 software. Relative quantification was calculated with the LightCycler^®^ 480 software based on the efficiency calibrated model [25]. Results are expressed as the ratio of expression of tested isolates *versus* the calibrator FR5133 (*ptx*P3 isolate producing both PRN and FHA) or the reference strain Tohama. Statistical analyses on the transcriptomic data were performed using the Wilcoxon rank-sum test on R software through the *stats* R package (version 3.1.2) [26]. Results with *p* < 0.05 were considered significant.

**Table 2:** qRT-PCR primers.

Primers Names	Primers Sequences 5' ≥ 3'
*recA-F*	TGGACGTGCAATACGC
*recA-R*	GACCATGCAGTTGGTG
*ptxA-F*	CCTACCAGAGCGAATATCTGGCAC
*ptxA-R*	GATTGGCGCGAGTCTGCT
*fhaB-F*	TCGGAGAGCCACAACT
*fhaB-R*	GTTCCGTATTGAAATTGAAGCC
*Prn-F*	CGGCGACCTTTACCCTTG
*Prn-R*	GGCTCCACTGCCCATTG
*cyaA-F*	GGTCAGCTATGCCGCCCT
*cyaA-R*	TTCTCCGTGCGCTTGCCGTA

### 2.6. Interaction with Human Tracheal Epithelial Cells

Epithelial cells growth: Cells of the human tracheal epithelial cell line (HTE) were plated in tissue culture trays coated with collagen G (Biochrom GmbH, Berlin, Germany) and cultured as previously described [17]. Bacterial cytotoxicity, invasion and persistence towards HTE cells were assessed as previously described [17]. Briefly, bacteria were added to cells at a 100:1 bacteria-to-cell ratio in 24-well plates. Plates were gently centrifuged and incubated at 37 °C, in the presence of 5% CO_2_ for 8 h. Cytotoxicity was determined every 2 h with the Cytotox96 assay kit (Promega, Madison, WI, USA) which measures the lactate dehydrogenase activity released into the medium. In each experiment, *B. bronchiseptica* RB50 was used as a positive control. For invasion assays, infected culture plates were incubated at 37 °C in the presence of 5% CO_2_ for 5 h. The cells were then washed extensively and incubated for two additional hours with 100 μg/mL gentamycin (Sigma-Aldrich France, St. Quentin Fallavier, France) to kill extracellular bacteria. Cells were then lysed with water and the number of intracellular *Bordetella* determined by CFU count after plating the lysate on BGA plates that were further incubated 3 days at 37 °C. For persistence assays, the concentration of gentamycin in the cell culture medium was reduced to 10 μg/mL after the initial 5 h of incubation and the infected cells were left to incubate for another 48 h. The number of intracellular *Bordetella* was then determined after cell lysis as described above. At least three independent assays were performed and each condition was ran in duplicate in two separate wells. Results are normalized *vs.* Tohama. Statistical analyses were obtained using a *t*-test and results with *p* < 0.05 were considered significant (Figure 4). 

## 3. Results

### 3.1. Whole Genome Data Analysis

The genome of the reference strain Tohama was re-sequenced and 22 SNP were found as compared to the published sequence. Analysis reported more than 200 SNPs in each of the sequenced isolate’s genome (Table 3) with a mean of 25% synonymous, 40% non-synonymous, 15% intragenic, 17% intergenic.

**Table 3:** Number of SNP detected after whole genome sequencing.

SNP details	Tohama (+)	CIP1672 (FHA−)	FR4624 (PRN−/FHA−)	FR4684 (PRN−)	FR4929 (+)	FR5133 (+)	FR5187 (PRN−)
SNP	22	205	252	268	266	279	286
Synonymous	4	54	67	69	70	75	78
Non synonymous	8	82	100	98	105	100	105
Intragenic	4	28	36	35	34	29	36
Stop	0	0	2	0	1	2	3
Intergenic	6	27	30	44	40	44	43
Frame shift	0	12	15	20	14	27	19
Insertion codon	0	2	2	2	2	2	2

**Figure 1:**
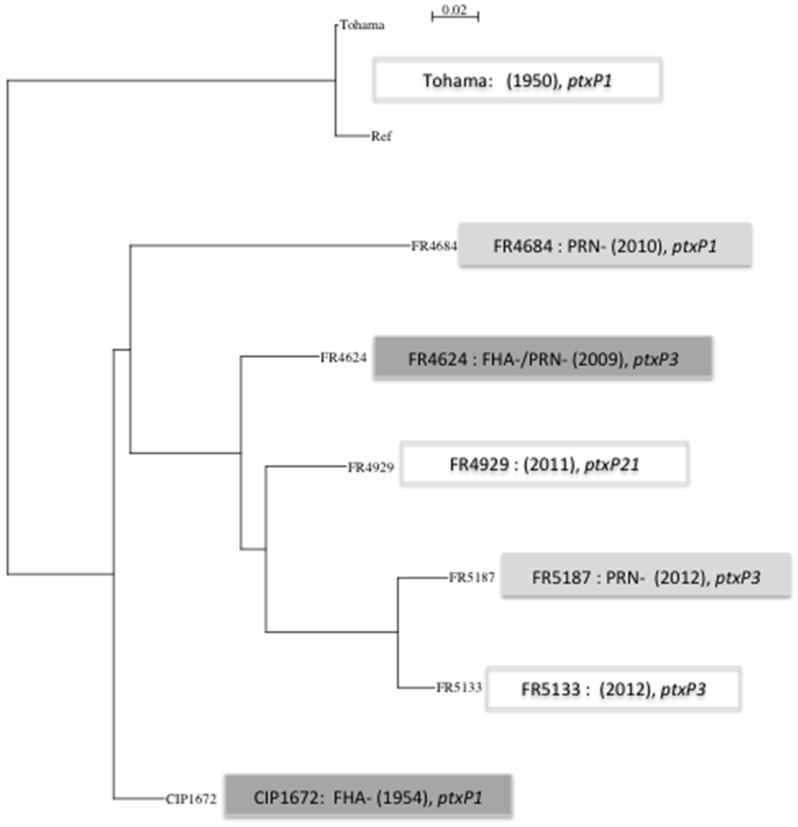
Phylogenetic tree based on 337 SNP. 337 SNP (comprising all SNPs found in all tested isolates) were concatenated using SynTview (http://genopole.pasteur.fr/SynTView/). Alignment was done using multalin (http://multalin.toulouse.inra.fr/multalin/) and the phylogenetic tree was done using Quicktree (http://mobyle.pasteur.fr/cgi-bin/portal.py?#forms::quicktree).

As expected, allelic variations were mostly observed in virulence-associated genes and in genes encoding membrane proteins for isolates producing all virulence factors. New features were however observed in vaccine antigen deficient isolates and in the two most recently collected ones (Table 4). SNPs were observed in the *ptxP* promoter sequence and within *ptxA* determining the classical corresponding alleles (*i.e.*, *ptxP1* or *ptxP3* and *ptxA-1* or *ptxA-2*). A new allele was observed for isolate FR4929 and was deposited in Genbank as *ptxP21* (Accesion Nb: KT036676). SNPs were also found within *ptxB* and *ptxC*. SNPs leading to PRN2/PRN1 alleles were observed within *prn.* Moreover, using sanger sequencing, we detected a deletion within the *prn* promoter of the PRN− isolate FR5187, a 84 bp deletion within the *prn* gene of the PRN− isolate FR4684 and an IS insertion (IS481) within the *prn* gene of the PRN− isolate FR4624 [27], responsible for their PRN phenotype. Deletions within the promoter of *fim2* (*pfim*) gene were observed in isolates non-producing Fim2 as evidenced using agglutination test [27]. In addition, a non-synonymous SNP is observed in *fim3* corresponding to allelic polymorphism (*i.e.*, *fim3-1*/*3-2*)*.* Other SNPs were found in other genes or promoter associated with pathogenicity: *bvgS*, *fhaS*, in some T3SS genes (*bscC*, *bscI*, *bopB*), *sphB1*, *lgmA*. In addition, the partial amplification of *fhaB* gene revealed the probable presence of an IS in the *fha*B gene of PRN-/FHA-FR4624 isolate and a deletion that leads to a premature stop codon within the *fhaB* gene for CIP1672 [27]. No SNPs were observed within *cya*A and *cya*C genes or their promoter. A phylogenetic tree based on 337 concatenated SNPs (representing all SNP identified in this study) illustrates that PRN− isolates do not segregate apart from PRN+ isolates of the same period and that the two most recent FR5133 and FR5187 isolates are close to FR4929, the *ptxP21* isolate and to the PRN−/FHA− deficient isolate FR4624 (Figure 1) while segregating together. 

**Table 4:** SNPs associated to virulence.

Tohama Reference Genome NC_02929 Position	AN in theRef.	Tohama *ptxP1*	CIP1672 (FHA-) *ptxP1*	FR4624 (FHA-/PRN-) *ptxP3*	FR4684 (PRN-) *ptxP1*	FR4929 *ptxP21*	FR5133 *ptxP3*	FR518 (PRN-) *ptxP3*	SNP Observed	*SNP Type*	*Gene or Promoter Localisation*	Nucleic Acid Change within Codon	AminoAcid Change
3988168	G	G	G	A	G	A	A	A	A	*intergenic*	*ptxp*		
3988244	G	G	G	G	G	A	G	G	A	*intergenic*	*ptxp*		
3988941	G	G	A	A	G	A	A	A	A	*non synonymous*	*ptxA*	atG/atA	M/I
3989239	G	G	A	A	A	A	A	A	A	*non synonymous*	*ptxB*	Ggc/Agc	G/S
3991376	C	C	C	T	C	T	T	T	T	*synonymous*	*ptxC*	tgC/tgT	
1098920	*	*	*	*	*	C	C	C	INS (C)	*frameshift*	*prn*	ggt/gCgt	G/A
1098922	G	G	G	G	G	T	T	T	T	*non synonymous*	*prn*	Gcg/Tcg	A/S
1098926	T	T	T	T	T	*	*	*	DEL (T)	*frameshift*	*prn*		
1098918	T	T	T	T	T	C	C	C	C	*synonymous*	*prn*	ggT/ggC	
1176534	*	G	G	G	*	G	G	G	INS (G)	*intergenic*	*pfim2*		
1176535	*	G	G	G	*	G	G	G	INS (G)	*intergenic*	*pfim2*		
1176536	*	G	G	G	*	G	G	G	INS (G)	*intergenic*	*pfim2*		
1176541	*	G	G	G	G	*	G	*	INS (G)	*intergenic*	*pfim2*		
1176542	*	G	G	G	G	*	*	*	INS (G)	*intergenic*	*pfim2*		
1176543	*	G	G	G	G	*	*	*	INS (G)	*intergenic*	*pfim2*		
1176544	*	G	G	G	G	*	*	*	INS (G)	*intergenic*	*pfim2*		
1176545	*	G	G	G	G	*	*	*	INS (G)	*intergenic*	*pfim2*		
1176546	*	A	A	A	A	*	*	*	INS (A)	*intergenic*	*pfim2*		
1626880	G	G	G	G	G	C	C	C	C	*intergenic*	*pfim2*		
1637246	C	C	C	C	T	C	C	C	T	*intergenic*	*pfim2*		
1647861	C	C	C	A	C	A	A	C	A	*non synonymous*	*fim3*	gCg/gAg	A/E
1984103	T	T	C	C	C	C	C	C	C	*non synonymous*	*fimD*	tTc/tCc	F/S
1965604	T	T	C	C	C	C	C	C	C	*non synonymous*	*bvgS*	Aag/Gag	K/E
1968699	G	G	G	A	G	G	G	G	A	*intergenic*	*pfhaB*		
2826237	G	G	G	G	G	A	A	G	A	*non synonymous*	*fhaS*	Cac/Tac	H/Y
223961	G	G	G	G	G	G	G	A	A	*non synonymous*	*sphB1*	Gta/Ata	V/I
224066	G	G	G	G	G	A	A	G	A	*non synonymous*	*sphB1*	Gcc/Acc	A/T
511992	A	A	A	G	A	G	G	G	G	*intergenic*	*pbteA*		
514171	G	G	G	A	G	A	A	A	A	*intergenic*	*pbteA*		
2374322	T	T	T	C	T	C	C	C	C	*non synonymous*	*bscI*	tAc/tGc	Y/C
2376650	G	G	G	A	G	A	A	A	A	*non synonymous*	*bopB*	Ccc/Tcc	P/S
2363842	C	C	C	C	T	C	C	C	T	*synonymous*	*bscC*	ctG/ctA	

* corresponds insertions or deletions.

### 3.2. Targeted Transcriptomic Analysis of cyaA, ptxA, fhaB and prn Expression

**Figure 2:**
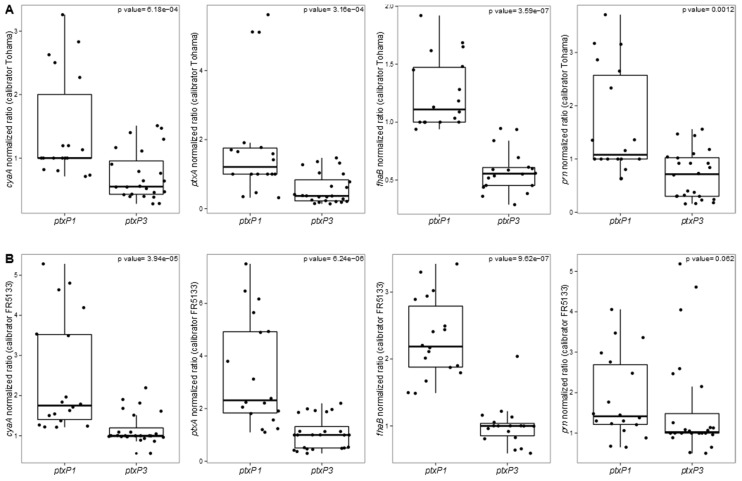
Box-plots of qRT-PCR data (based on liquid cultures). Comparison of the expression levels of the key virulence genes *cyaA*, *ptxA*, *fhaB*, and *prn* between *ptxP1* and *ptxP3* isolates. Data have been normalized *vs.* (**A**) the *ptxP1* calibrator Tohama or (**B**) the *ptxP3* calibrator FR5133. Dots represent each single experiment performed. For each isolate, three technical replicates from each of the two biological replicates are presented. Exact p-values reported for each comparison.

Using qRT-PCR, we compared the gene expression of the key virulence genes *ptxA*, *cyaA*, *prn*, and *fhaB* in isolates producing or not PRN or FHA in a genotypic *ptxP1* and *ptxP3* background (*ptxP21* was included for this analysis within *ptxP3* group). We discovered a significant reduction of the transcriptional levels of *ptxA*, *cyaA*, and *fhaB* between *ptxP1* isolates and *ptxP3/ptxP21* isolates with both the calibrators taken into account (*i.e.*, Tohama as reference for the *ptxP1* isolates and FR5133 as reference for the *ptxP3* isolates) (*p* < 0.005, Wilcoxon rank-sum test) while we found significant differences between *ptxP1* and *ptxP3* isolates in *prn* expression only when we used the reference strain Tohama as calibrator (*p* < 0.005, Wilcoxon rank-sum test) (Figure 2).

**Figure 3:**
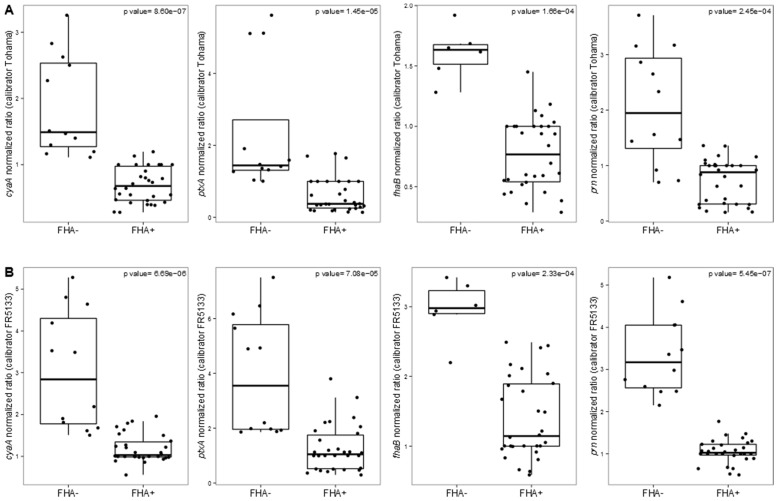
Box-plots of qRT-PCR data (based on liquid cultures). Comparison of the expression levels of the key virulence genes *cyaA*, *ptxA*, *fhaB*, and *prn* between FHA− and FHA+ isolates. Data have been normalized *vs.* (**A**) the *ptxP1* calibrator Tohama or (**B**) the *ptxP3* calibrator FR5133. Dots represent each single experiment performed. For each isolate, thrree technical replicates from each of the two biological replicates are presented. Exact *p*-values reported for each comparison.

Since the genetic background of an isolate could deeply affect its gene expression, we compared the transcriptomic levels of *ptxA*, *cyaA*, *prn*, and *fhaB* in isolates producing or not PRN or FHA. We discovered that FHA− isolates present a significantly higher expression of *ptxA*, *cyaA*, and *prn* compared to isolates producing all virulence proteins using both the calibrators (Figure 3) (*p* < 0.001, Wilcoxon rank-sum test). Noteworthy, we included in the analysis on *fhaB* expression also the FHA−, *ptxP1* isolate CIP1672 since it is able to produce observable levels of *fhaB* transcripts (but it does not produce FHA due to the presence of a stop codon in *fhaB*). FR4624 was not included in the analysis because *fhaB* is not at all expressed (probably due to an IS within *fhaB*). No significant differences in *ptxA*, *cyaA*, *prn*, and *fhaB* gene expression have been observed between PRN− and PRN+ isolates.

### 3.3. Properties toward Human Tracheal Epithelial Cells

**Figure 4:**
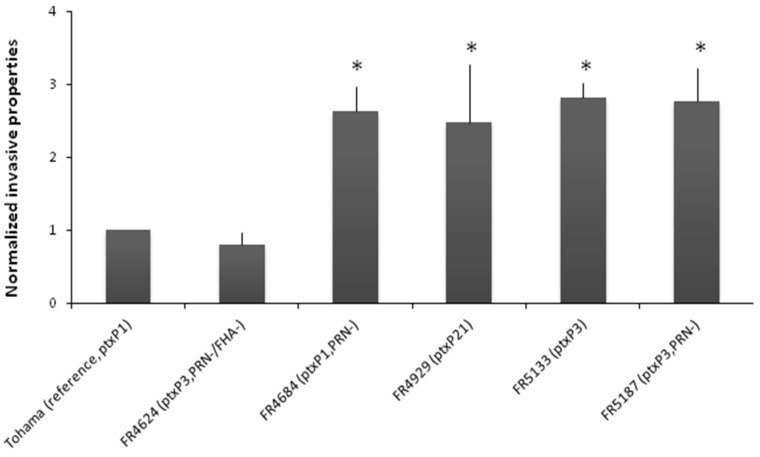
Normalized invasive properties of tested isolates *vs.* Tohama. At least three independent assays were done, each in duplicate. Results are normalized *vs.* Tohama. Statistical analysis were obtained using a *t*-test and results with *p* < 0.05 were considered significant (*).

None of the tested *B. pertussis* isolates were cytotoxic for HTE cells. Surprisingly, though, all isolates were shown significantly more invasive than the reference strain Tohama, regardless of the production of PRN (Figure 4) with the exception of FR4624 (PRN−/FHA−) which was, as expected, less invasive. For all tested isolates no multiplication was observed within epithelial cells and only less than 0.5% were able to persist 24 h within cells [27]. 

## 4. Discussion

*B. pertussis*, the agent of whooping cough, evolved from a human-associated lineage of *B. bronchiseptica*, the mammalian pathogen ([5,6]). According to recent genomic analysis of more than 300 isolates, it was estimated that *B. pertussis* species emerged around 500 years ago [15]. The adaptation of *B. pertussis* to its human host proceeded mostly through gene loss but also through other mechanisms such as point mutations and horizontal gene transfer in loci associated with virulence as loss of the *O*-antigen or acquisition of pertussis toxin ([14,15,28]). However, *B. pertussis* more recently had to face vaccine-induced immunological pressure. First wP vaccine pressure during 50–60 years in developed countries and then aP vaccine pressure for the past 10–15 years. It was observed in the mid-1990s that isolates circulating in high and low coverage regions were different and also that such isolates circulating in high vaccine coverage regions were different from the vaccine strains used for the production of wP vaccines ([1,29]).

Recent development of WGS allowed fine analysis of SNPs in the *B. pertussis* genome of circulating isolates ([12,13,14,15]). The mean SNP density has been evaluated to be 0.0013 SNP/bp [15] based on the sequence of more than 300 isolates collected before 2010 when compared to the Tohama reference strain, leading to the identification of a total of 5414 SNPs. According to these SNPs, isolates were segregated in two main groups. The first one gathered few isolates close to *B. pertussis* 18323, an isolate with particular features, some of them being closer to *B. bronchiseptica* than to *B. pertussis*. The majority of the other *B. pertussis* isolates were found in the second group where several periods of increase in diversity were observed, the last one corresponding to *ptxP3* and *fim3-2* isolates emergence that occured during the wP vaccine era (*i.e.*, before aP vaccine introduction) [15]. Recently, Sealey *et al.* [30] analyzed isolates circulating during the last cycle of pertussis in United Kingdom. They observed that aP vaccines antigen-encoding genes are evolving at a higher rate than other genes but this was true even prior to the introduction of wP and aP vaccines. However, in their study only one isolate deficient in the production of PRN was analyzed. Our genomic analysis including PRN- and FHA-deficient isolates, compared to the Tohama strain, revealed a mean of 270 SNPs (*i.e.*, 0,000066SNP/bp when referring to Tohama Reference genome size) most of them were already detected previously ([13,14,15,31]). We found that the two most recent *ptx*P3 isolates FR5133 and FR5187 are close to the *ptx*P21 isolate FR4929 sharing SNP within *prn*, *pfim2*, and *ptx*C, but also within T3SS genes, *bsc*I, and *Bop*B, or within the *btcA*/*bteA* promoter region (Table 4). 

We noticed that PRN− or FHA− isolates mostly harbor a *fim3-1* allele and this was confirmed using PCR on additional PRN-deficient isolates [27]. In Canada [32], and in different European countries [33], PRN-deficient isolates have been reported to be either *fim3-1* or *fim3-2*, but in Washington state, most of isolates collected during the late epidemics were PRN-deficient (76%) and *fim3-1* [34]. Are PRN− isolates more often associated with *fim3-1* allele? In a retrospective study of US Pertussis isolates collected from 1935 to 2009, the increase in *fim3-2* was associated with an increase in U.S. Pertussis notifications observed since 2000 [35]. Further evolution of *fim3* gene and Fim production should be investigated.

When considering PRN− or FHA− isolates, we found few specific SNPs besides those leading to the non-production of PRN or FHA. To be noticed in the PRN− isolate FR5187, a non-synonymous SNP within the auto-transporter SphB1 encoding gene that is found in none of the other isolates but that was previously reported in a recent study on 2 other French PRN+ isolates collected in 2005 (FR3407) and in 2007 (FR3713). This SNP leading to a V/I AA_120_ modification within SphB1 can, therefore, not be related to the PRN− phenotype of FR5187 isolate [15] but might have an impact on these isolates phenotype *in vivo*.

As in other studies ([15,30]), *ptxP3* is the predominant allele of isolates circulating in highly aP vaccinated countries but few isolates are characterized by other *pt*xP alleles as *ptxP21* for FR4929 or *ptxP1* for FR4684. When considering the *ptxP21* isolate FR4929, it is interesting to observe that it is more closely related to *ptxP3* isolates than to *ptxP1* ones. Indeed *ptxP21* allele (as *ptxP15*, *ptxP18*, *ptxP19*, and *ptxP20*) presents a common SNP with *ptxP3* isolates in position 3988168 (position based on Tohama reference) and one additional SNP that is different for each of these alleles. All these new alleles could, therefore, be considered as variants of *ptxP3*. Concerning AC-Hly, no polymorphism has been found within *cyaA* in all tested isolates and it is expressed similarly by all isolates except in FHA-deficient ones (this study and King [36]).

If massive genomic data were published in databases in the last decade, few transcriptomic ones are available concerning *B. pertussis* isolates, in particular concerning vaccine antigen deficient isolates. Our aim was to perform a comparison with isolates harboring different *ptx*P alleles and producing or not PRN and/or FHA. Our transcriptomic data revealed that *ptxP3* isolates as the *ptxP21* one present reduced transcriptional levels of the key virulence genes *cyaA*, *ptxA*, *prn*, and *fhaB* compared to *ptxP1* isolates (Figure 2). Comparing the transcriptional profiles on a genome-wide scale of *ptx*P1 and *ptx*P3 isolates producing all virulence proteins, King and colleagues [37] reported that the transcriptional fold changes of genes associated with virulence were lower than 1.7 fold in *ptxP1* isolates compared to *ptxP3* isolates, except for *fim*2 and *fim*3. Similarly, de Gouw and colleagues [38], comparing the expression profiles of virulence-associated genes between a *ptxP1* and a *ptxP3* isolate both collected in 2000 in the Netherlands found only two genes with significant higher level of expression (three-fold) in the *ptxP3* isolate. No higher expression of *ptx* or *ptl* genes (corresponding respectively to pertussis toxin subunits and secretion genes) was reported in these two studies suggesting no higher production of PT by *ptxP3* isolates [39]. On the contrary, we discovered that, in our *in vitro* conditions, the French *ptxP3* isolates express lower levels of *ptxA* compared to *ptxP1* isolates.

When considering PRN− isolates, we didn’t observe any significant differences in the expression levels of *cyaA*, *ptxA*, *prn* and *fhaB* between *ptxP1* and *ptxP3* isolates. Such observations in PRN-deficient isolates fit with *in vitro* and *in vivo* studies that reported PRN− isolates to be as virulent as PRN+ ones [8,18].

The data obtained from the transcriptional analysis of FHA-deficient isolates were surprising. Such isolates are rare as compared to PRN-deficient isolates [16]. None have been found in the USA or Australia, so far, according to published data, although these countries are facing the highest incidence of PRN-deficient isolates. However, their surveillance is very important. In fact, PRN-deficient isolates were very rare during the pre-vaccine and the post-wP vaccine era but are now increasingly represented among collected isolates. Great attention should also be given to FHA-deficient isolates which population might undergo the same fate as PRN-deficient isolates. Interestingly, these isolates present a different transcriptomic behavior since the expression of *ptx*A, *cya*A or *prn* genes is significantly increased as compared to isolates producing FHA. FHA plays an important role in *B. pertussis* virulence and it is involved in cell adhesion as confirmed by our results with HTE cells. Could these results be a consequence of a different regulation? The *bvg* regulatory system is known to control the expression of a large number of genes [40], but no sequence difference was found in the *bvg* operon for FHA-deficient isolates. Hfq is a RNA chaperone that has been shown to be required for *B. pertussis* virulence and Tohama ΔHfq produces less AC-Hly and secrete less PT [41]. Furthermore, loss of Hfq has been reported to have a deep impact on expression of several virulence factors, like some type-three-secretion system genes, *vag8*, *brkA*, *tcfA* or *ptx*/*ptl* locus [42]. We observed no SNP within *hfq* gene for FHA-deficient isolates. However, in these isolates, the absence of FHA mRNA and consequently the non-production of FHA protein, might lead to a higher hfq-mediated transcriptomic expression of other virulence genes such as *prn*, *cyaA* or *ptxA*. Similarly, small noncoding regulatory RNAs (sRNA) might also increase the expression of virulence genes in absence of FHA mRNA [43]. More investigations are needed to better understand the differential gene expression observed in FHA-deficient isolates.

In addition to these genomic and transcriptomic observations, we evaluated the cytotoxic, invasive, and persistence properties of isolates producing or not PRN towards HTE cells. PRN is an auto-transporter playing a role in adhesion through different binding sites and in particular its RGD motif [44]. This motif seems to be a key component in bacteria to cell interactions for *B. pertussis* in various models ([45,46]). However, its real implication in cell adhesion, and thus in invasive properties, is still not well understood since conflicting results have been reported in different studies carried out with *B. pertussis* Tohama Δ*prn* constructed mutants and different cell lines ([46,47,48]). PRN-deficient isolates were shown to be more invasive in human monocyte-derived dendritic cells [46] or in HTE cells [17] when compared respectively to the reference strains 18323 or Tohama. Our present data lead to similar conclusions. Nevertheless, the two recent isolates producing PRN (FR4929 and FR5133) are also found more invasive than the reference strain Tohama. Isolates currently circulating in France, a region using aP vaccines for more than 13 years, may thus have a better fitness for invading epithelial cells whatever their PRN phenotype and loss of that adhesin is probably not the only explanation of the observed increased invasiveness as previously thought [17]. Moreover, as Lamberti and colleagues [48] who reported that intracellular *B. pertussis* never replicated within epithelial respiratory cells even if remaining viable, we didn’t observe multiplication of the isolates within the HTE cells.

All the studies performed since the discovery of *B. pertussis* by O. Gengou and J. Bordet [49] showed that *B. pertussis* population is monomorphic but able to adapt quickly to new situations, such as variations in herd immunity. Pertussis was known to be cyclical disease during the pre-vaccine era and remains as such even in regions with high aP vaccine coverage (not only in young children but also in adolescents, and now more and more in adults). This characteristic indicates that the vaccines (wP or aP) are protective against severe disease in infants but not against the carriage or transmission of the bacterium in the population, particularly in individuals with waning immunity or in possessing different immunity related to their vaccine or disease history.

Recently, using the baboon model, it was shown that aP vaccines were protecting even less against transmission than the wP vaccine [50] and this needs to be confirmed in the human population. In developed countries herd immunity is driven by individuals who either (i) presented clinical manifestation of Pertussis and who received or not a vaccine booster; (ii) received a primary wP vaccination and aP boosters; or (iii) received a primary aP vaccination and aP boosters. It is now known that immunities induced by aP and wP vaccines are different [51], the herd immunity is, thus, constantly evolving with vaccine strategies. In turn the bacterium needs to adapt regularly to these changes. Recently, in regions using aP vaccines since 12–15 years, a pertussis cycle occurred with an increase of PRN− isolates. The proportion of these PRN− isolates varied as well as the intensity of the cycle [16]. We showed that these PRN− have a better fitness in an aP vaccinated background using the murine model [19], observation which was recently confirmed [20]. The vaccine coverage may be the key parameter leading to these observations. Indeed, the regions with the highest proportion of PRN− isolates were also those with either a lower vaccine coverage, that had suppressed a vaccine booster, or without any vaccine booster. The prevalence of bacteria presenting a better fitness in a given population can thus be expected to increase each time the proportion of susceptible is increasing or each time a major change in herd immunity occurs. It is then of high importance (i) to establish a surveillance of the disease in different regions of the world using different type of pertussis vaccine and different coverage, and (ii) to continue to isolate the bacterium. The murine model was shown to be very effective in demonstrating a better fitness of *B. pertussis* isolates. It enabled to show that, depending on the wP vaccine used, *B. pertussis* isolates harboring *prn*3 or *prn*2 alleles had different fitness than isolates harboring *prn*1 ([36,52,53]. Recently, still using the same model, PRN− isolates were shown to have a better fitness in an aP vaccinated background [19]. The cytotoxicity and the invasiveness models were also shown to be useful to observe differences between isolates carrying different alleles or non-producing some antigens ([19]; this study). It is then of high importance, as shown in this study, to analyze the isolates using not only WGS but also transcriptomic, proteomic, animal, and cellular models and to do clinical studies such as transmission and carriage in order to identify quickly the changes occurring in the next cycle and to better understand the circulation of *B. pertussis* in the population.

## 5. Conclusions

In conclusions, in the present study, we were able to demonstrate differences between the expression of virulence factors in FHA deficient *B. pertussis* isolates presently circulating in regions of the world routinely using aP vaccines since one or two decades. Furthermore, we showed that the isolates producing or non-producing vaccine antigens, circulating in these regions, are more invasive in HTE cells. All these observations need further investigations and confirm the importance to use different tools to analyze the adaptation of *B. pertussis* to herd immunity. 
